# Supplementary material for: The Specificity of Motor Learning Tasks Determines the Kind of Skating Skill Development in Older School-Age Children
Source: Sports (Basel). 2020 Sep 14;8(9):126. doi: 10.3390/sports8090126 (PMC7552761; doi:10.3390/sports8090126)
Supplement: Supplementary file 1 [file sports-08-00126-s001.pdf]

sports

MDPI

Supplementary Material

The Specificity of Motor Learning Tasks Determines the Kind of Skating Skill Development in Older School-Age Children

Dominik Novak [1,*], Adam Tomasek [1], Patrycja Lipinska [2], Petr Stastny [1,*]

Table S1. The test results in study measurement.

Test	Pre (mean± SD)	Post 1 (mean± SD)	Post 2 (mean± SD)
1. Agility without a puck (s)	16.33 ± 0.40	16.25 ± 0.28	15.94 ± 0.43
2. Agility with a puck (s)	18.02 ± 0.98	17.61 ± 0.89	17.27 ± 0.87
3. Straight 30 m sprint without a puck (s)	6.39 ± 0.35	6.14 ± 0.18	5.98 ± 0.19
4. Straight 30 m sprint with a puck (s)	6.39 ± 0.34	6.46 ± 0.26	6.11 ± 0.18
5. Backward skating for 30 m without a puck (s)	7.77 ± 0.83	7.83 ± 0.59	7.63 ± 0.52
6. Straight 4 m sprint speed without a puck (s)	1.93 ± 0.11	1.70 ± 0.12	1.68 ± 0.12
7. Straight 4 m sprint speed with a puck (s)	2.02 ± 0.10	1.69 ± 0.11	1.71 ± 0.10
8. Backward 4 m sprint speed without a puck (s)	2.37 ± 0.13	2.01 ± 0.13	2.03 ± 0.13

© 2020 by the authors. Submitted for possible open access publication under the terms and conditions of the Creative Commons Attribution (CC BY) license (http://creativecommons.org/licenses/by/4.0/).
